# Use of High-Resolution Multispectral UAVs to Calculate Projected Ground Area in *Corylus avellana* L. Tree Orchard

**DOI:** 10.3390/s22197103

**Published:** 2022-09-20

**Authors:** Gessica Altieri, Angela Maffia, Vittoria Pastore, Mariana Amato, Giuseppe Celano

**Affiliations:** 1 School of Agriculture, Forestry, Food and Environmental Sciences, University of Basilicata, 85100 Potenza, Italy; 2 Agriculture Department, Mediterranea University, 89124 Reggio Calabria, Italy; 3 AGES s.r.l., University of Basilicata, 85100 Potenza, Italy; 4 Department of Pharmacy, University of Salerno, 84084 Fisciano, Italy

**Keywords:** precision agriculture, hazelnut trees, irrigation strategies, UAV multispectral images, NDVI, CHM, PGA

## Abstract

In the last decade, research on *Corylus avellana* has focused on improving field techniques and hazelnut quality; however, climatic change and sustainability goals call for new agronomic management strategies. Precision management technologies could help improve resource use efficiency and increase grower income, but research on remote sensing systems and especially on drone devices is still limited. Therefore, the hazelnut is still linked to production techniques far from the so-called Agriculture 4.0. Unmanned aerial vehicles platforms are becoming increasingly available to satisfy the demand for rapid real-time monitoring for orchard management at spatial, spectral, and temporal resolutions, addressing the analysis of geometric traits such as canopy volume and area and vegetation indices. The objective of this study is to define a rapid procedure to calculate geometric parameters of the canopy, such as canopy area and height, by methods using NDVI and CHM values derived from UAV images. This procedure was tested on the young *Corylus avellana* tree to manage a hazelnut orchard in the early years of cultivation. The study area is a hazelnut orchard (6.68 ha), located in Bernalda, Italy. The survey was conducted in a six-year-old irrigated hazelnut orchard of Tonda di Giffoni and Nocchione varieties using multispectral UAV. We determined the Projected Ground Area and, on the *Corylus avellana* canopy trough, the vigor index NDVI (Normalized Difference Vegetation Index) and the CHM (Canopy Height Model), which were used to define the canopy and to calculate the tree crown area. The projection of the canopy area to the ground measured with NDVI values > 0.30 and NDVI values > 0.35 and compared with CHM measurements showed a statistically significant linear regression, R^2^ = 0.69 and R^2^ = 0.70, respectively. The ultra-high-resolution imagery collected with the UAV system helped identify and define each tree crown individually from the background (bare soil and grass cover). Future developments are the construction of reliable relationships between the vigor index NDVI and the Leaf Area Index (LAI), as well as the evaluation of their spatial-temporal evolution.

## 1. Introduction

The European hazelnut (*Corylus avellana* L.) is one of the most important species in the world in the category of nuts [1,2]. Hazelnut tree irrigation is an emerging management issue. Orchard water supplies have to satisfy the realistic demands of each tree species [3] and, for the hazelnut tree, the crucial period for water demand is from May to September [4]. In traditional areas of production, an average of 800–1000 mm of precipitation is needed, regularly distributed throughout the year [5]. In the Mediterranean basin, the current average annual temperature is 1.4 °C higher than in the late 19th century and the largest differences between the two periods are recorded during the summer. Climate change, now underway, has already stressed significant vulnerability and susceptibility of some agroecosystems. For every 1 °C of global warming, the average precipitation is likely to decrease by about 4% in most regions and particularly in areas in the center and the south of Italy [6].

Drought, excessive heat load, and high daily irradiation are considered limiting factors for agricultural production, with consequences on plant growth resulting in low productivity and fruit quality. Water stress in the hazelnut tree, *Corylus avellana*, adversely affects fruit production and quality, causing premature cessation of fruit growth, early leaf fall, a larger proportion of empty fruits, and increased susceptibility to diseases [7,8]. Recent studies have documented the impact of changes in temperature and precipitation on the hazelnut tree. Increasing the number of days with maximum temperature above 35 °C and relative humidity below 70% causes severe water stress, resulting in yield decline and reduced vegetative growth, combined with a reduction in nucule filling [9,10]. This is more evident for the Tonda di Giffoni variety of hazelnut, highly appreciated for its processing quality. 

The interest in hazelnut cultivation is also linked to its important role in the rural landscape of some areas of southern Italy. In fact, the planting of more sustainable crops such as hazelnut plants can be a key strategy in preventing soil erosion and land degradation [11].

The advantages of remote sensing are highlighted in several works. It is fast, non-destructive, and can study a large area of detection with the possibility of quantifying biophysical parameters [12,13]. Although it is widely used in the scientific research community, it has limitations; yet, in recent years, UAVs (Unmanned Aerial Vehicles) are preferred by agricultural operators [14]. UAVs, usually called drones, are aircrafts without a human pilot on board. UAVs are controlled by an operator on the ground [15]. One limitation of multi-rotor UAVs is battery duration, although this enables UAVs to operate in relatively small-to-medium-sized orchards [16]. 

UAVs are becoming increasingly available to satisfy the demand for rapid real-time monitoring of orchard management at spatial, spectral, and temporal resolutions adapted to analyze geometric traits such as canopy volume and area [14,16,17,18]. Spatial resolution is a measure of the smallest detectable object on the ground. The number of pixels used for image building in the sensor itself, and its distance from the ground, help to determine the size of the pixels on the ground and the resulting image footprint [3]. In addition, data acquisition is fast and images are georeferenced [19]. The mission flight setting depends on the characteristics of the case study: the size of the area, the flight autonomy, and the sensor features of the UAV that influence the Ground Sample Distance (GSD) and the image resolution. 

Data produced from the use of UAVs and the photogrammetric processing allow to create orthorectified mosaics (orthophoto mosaics), digital elevation models (DEMs), three-dimensional (3D) point clouds, and vegetation indices. Therefore, vegetation monitoring is possible because these kinds of outputs allow vegetation detection and feature extraction such as tree height, projected ground area (PGA), diameter, canopy volume, and individual tree counts [15,17]. In the last decade, research on *Corylus avellana* has focused on the improvement of field management and on the quality of products; however, techniques are lagging behind advanced agronomic practices. Research works involving the use of environmental remote sensing systems are very few and the review of Zhang et al. [16] reports no published paper on the use of UAVs in *Corylus avellana*. This parallels production techniques, which are far from exploiting available technology (e.g., the so-called Agriculture 4.0) [20]. Instead, precision management could help improve resource use and increase grower income [3].

The irrigation management of trees can be greatly aided by biometric data related to evapotranspiration such as leaf area and canopy geometry. In order to delimit individual parameters, each tree must be separated from background bare soil and grass cover. Several researchers’ increasing interest has focused on the extraction of tree canopies of different crops from UAV images in precision agriculture applications [17,21,22]. Marques et al. [4] propose to use the CHM (Canopy Height Model) or vegetation indices such as NDVI (Normal Difference Vegetation Index) as inputs into an image processing method to give an estimate of geometric traits in chestnut trees, such as canopy and diameter area and tree height. The individual tree detection can be run with acceptable accuracy from UAV derived CHMs [23]. The relationship between canopy cover and NDVI was shown by Tenreiro et al. [24] for various crop types, albeit at different levels of accuracy. Caruso et al. [25] highlighted the reliability of UAV imagery in estimating the geometric features of the olive tree, such as canopy volume and projected canopy area.

The Leaf Area Index (LAI) is among the most important biophysical variables of vegetation and represents the ratio between the total leaf area and the canopy projection area on the ground. LAI monitoring in the different phenological stages of the hazelnut tree allows the implementation of specific irrigation schedules. For the determination of LAI, it is necessary to calculate the PGA (Projected Ground Area) of the canopy and the leaf area. 

Accordingly, the objective of this study is to define a rapid procedure to calculate important geometric parameters of the canopy, such as canopy area and height, by methods using NDVI and CHM values derived from UAV images. This procedure has been tested on the young *Corylus avellana* tree to manage the hazelnut orchard in the early years of cultivation.

## 2. Materials and Methods

### 2.1. Experimental Site

**Figure 1:**
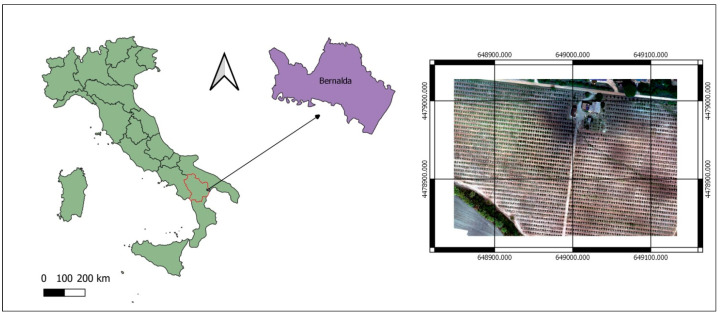
**Left**: geographical location of the farm, located in southern Italy; **right**: aerial photo of the research field taken from UAV with 6.29 cm/pixel.

The study area is a hazelnut orchard (6.68 ha), located in Bernalda, Italy (40°26.847′ N 16°45.420′ E Figure 1), with an elevation of 84 m above sea level.

The flight mission was conducted in a six-year-old irrigated hazelnut orchard of Tonda di Giffoni and Nocchione varieties. This latter represents the pollinator with 10% of the total number of plants. The plant density was 666 trees/ha, (tree spacing 5 m × 3 m) and the training system was multistemmed shrubs or multisystem bushes, designed with the aim of a classic growing system but with a greater number of plants per hectare.

### 2.2. UAV-Based Data Acquisition

Remotely sensed data were acquired with a multi-rotor DJI Phantom 4 (P4) Multispectral UAV, a high precision drone for multispectral imaging functions. The imaging system was equipped with six CMOS (complementary metal oxide semiconductor) cameras, including one RGB camera for visible light imaging in JPEG format and five monochrome cameras for multispectral imaging in TIFF format. Each sensor has 2.08 MP effective (2.12 gross MP), focal length of lens 5.74 mm, image size in width and height 1600 × 1300, and size lens in width and height 4.96 and 3.72 mm, respectively.

The five cameras acquire red, green, blue, red edge, and near infrared in the following imaging bands: Blue (B): 450 nm ± 16 nm; Green (G): 560 nm ± 16 nm; Red (R) 650 nm ± 6 nm; Red Edge 730 nm ± 16 nm; Near Infrared (NIR): 840 nm ± 26 nm.

The aircraft has an onboard RTK positioning system that provides centimeter-level accuracy.

The flight mission was conducted on 20 September 2021 in a grid pattern, nadir-oriented camera, at 120 m height, with a front and side imagery overlap of 70%. The total number of pictures acquired was 594, with a spatial resolution of 0.0611 m/pixel in a flight time of 12′48′′.

### 2.3. Image Processing Methods

**Figure 2:**
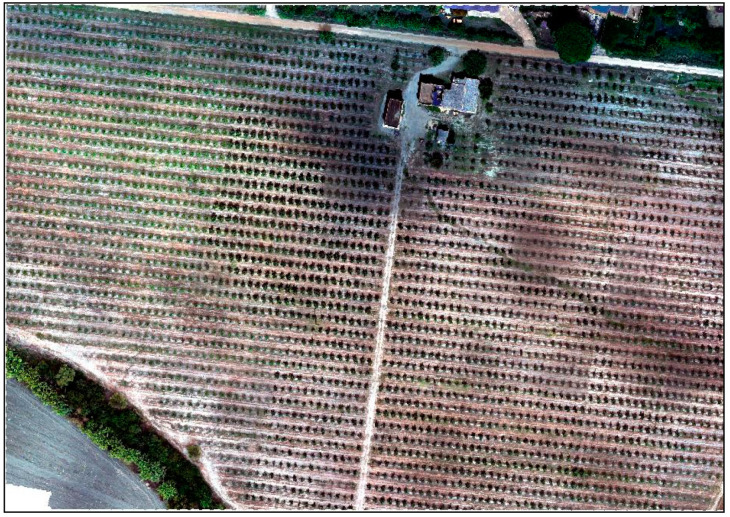
Orthophoto mosaic from aerial multispectral imagery collected using UAVs.

Photogrammetric processing of the acquired UAV imagery was carried out using Agisoft Metashape Professional software version 1.8.2 (Agisoft LLC, St. Petersburg, Russia). The processing steps were as follows: adding photos from the multi-camera system only in Tiff format; calibrating reflectance; aligning photos based on matching points between images; optimizing cameras (to improve the accuracy of the alignment for the RTK drone in a project without using GCP); building dense cloud; building DEM (Digital Elevation Model); building ortho-mosaic (Figure 2).

The output obtained from the image processing in the Agisoft Metashape software was a digital surface model (DSM).

Georeferenced and orthorectified images and DSM were exported in QGIS software version 3.22.4-Białowieża (Free Software Foundation, Inc., 51 Franklin Street, Fifth Floor, Boston, MA 02110-1301 USA) to be processed to calculate the Normalized Difference Vegetation Index (NDVI) [26] and the Canopy Height Model (CHM). The spatial resolution of all layers is 0.0611 m/pixel.

### 2.4. Canopy Delimited with NDVI and CHM Methods

The projected ground area (PGA) of the canopy (tree crown area) was measured with two methods.

#### 2.4.1. NDVI 

**Figure 3:**
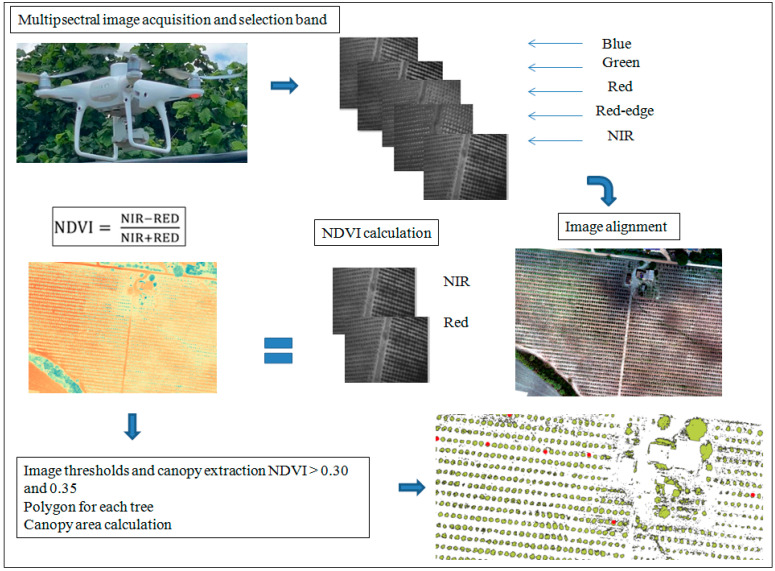
Flowchart of the procedure to delimit the hazelnut canopy using multispectral images acquired from a UAV.

Figure 3 shows the flow chart of the procedure to delimit the hazelnut canopy using multispectral images acquired from a UAV.

The projected area of the canopy was delimited, based on the spatial distribution of the NDVI values:

NDVI = NIR − RED/NIR + RED

where NIR and RED are the values of the reflectance in the near-infrared (840 ± 26 nm) and red (650 ± 6 nm) bands, respectively.

The NDVI values of bare and grassed ground were associated with pixel values in the areas between rows not affected by tree canopies. Pixels with NDVI values between 0.1–0.32 belonged to bare and grassed ground. NDVI values between 0.3–3.5 were also associated with pixels with DSM − DTM ≥ 0.40 m of the tree canopies. Therefore, we decided to test the NDVI threshold values of 0.3 and 0.35 to select the most suitable for tree discrimination. We applied the raster calculator to the whole investigated area, using the NDVI threshold values of 0.30 and 0.35. Seventy-five trees were randomly selected within the raster, showing only NDVI values ≥ 0.30 and 0.35, respectively. The single area was calculated and associated with the tree canopy value.

#### 2.4.2. Canopy Height Model (CHM) Method

From the DSM obtained with Agisoft Metashape, three lines parallel to the row and placed one meter apart were drawn in inter-row zones without canopy cover with the “advanced digitization panel tool”. Using the “points along geometry” command, measurement points were created at distances of three meters and staggered between the lines. Using the “Point Sampling Tool” (PST) plugin, the corresponding elevation values from the DSM raster were extracted at each point. Interpolation of the extracted elevation values was obtained using the “Triangulated Irregular Network (TIN) tool”. The CHM was determined by subtracting the DTM from the DSM with the raster calculator.

All processing was performed with the open-source QGIS software version 3.22.4-Białowieża (Free Software Foundation, Boston, MA, USA).

### 2.5. Leaf Area Measurement

The leaf area of the hazelnut trees of the Tonda di Giffoni variety was measured destructively on 10 suckers taken from 10 different hazelnut plants. The circumference at 20 cm above the ground was measured on the same suckers with a centimeter ribbon, and the number of leaves was counted. The leaf area was measured using ImageJ 1.53k software (Wayne Rasband and contributors National Istitutes of Health, USA) which delineates leaves with color contrast.

### 2.6. Statistical Analysis

Statistical analysis was carried out using RStudio software version 1.4.1103.0 (RStudio, PBC, Boston, MA, USA). Statistical descriptive and regression analyses were performed.

Pairs of CHM–NDVI data were divided into two data sets: data pairs were randomly assigned to Dataset I or II. Dataset I consisted of 50 values and Dataset II comprised 25 values. Dataset I was used for regression analysis to determine the best empirical model relating the CHM and NDVI values. Regression models were compared based on the minimization of the sum of the square residuals. The regression of CHM on NDVI from Dataset I was tested on Dataset II to estimate NDVIe values. The NDVIe values of Dataset II were then regressed on the measured NDVI values (NDVIm), and the NDVIe/NDVIm pairs were compared by the Wilcoxon signed-rank test.

In addition, the allometric relationship between the leaf area and the basal diameter of the suckers was constructed by linear regression analysis. 

## 3. Results

### 3.1. CHM/NDVI Method

**Figure 4:**
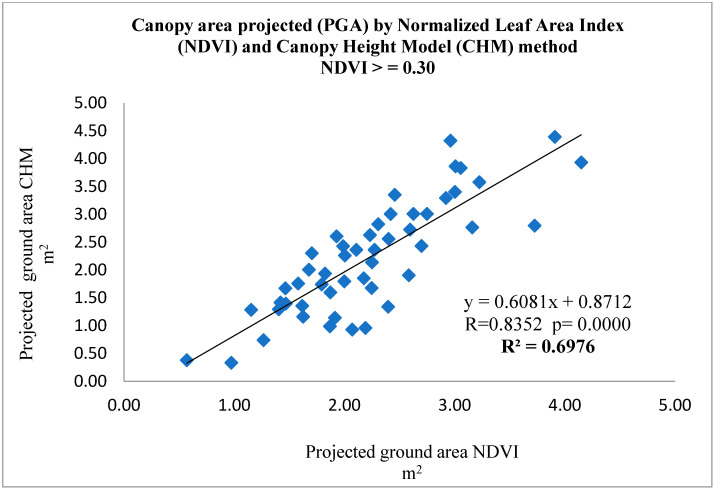
Relationship between PGA_CHM_ vs. PGA_NDVI_ with NDVI values > 0.30.

**Figure 5:**
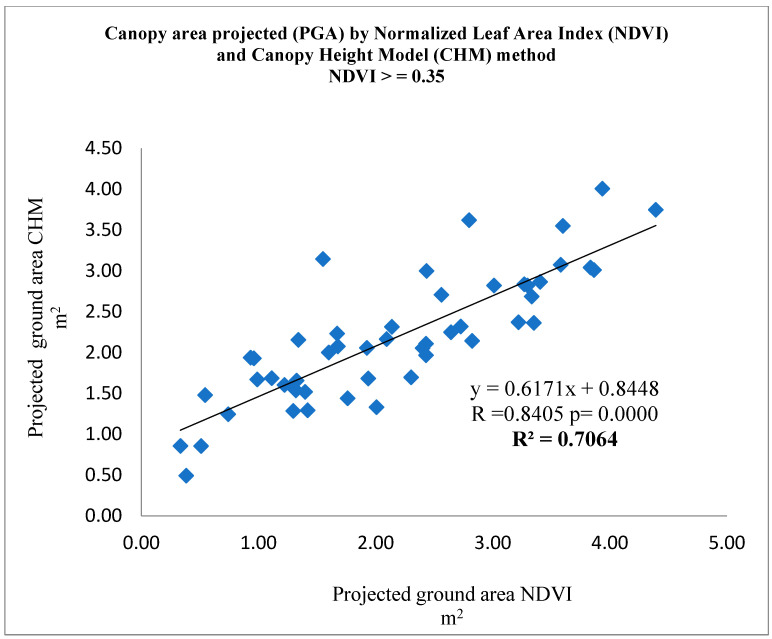
Relationship between PGA_CHM_ vs. PGA_NDVI_ with NDVI values > 0.35.

The projection of the canopy area to the ground measured with NDVI values > 0.30 and NDVI values > 0.35 compared with the CHM measurements showed a statistically significant (*p* < 0.0001) linear regression (R^2^ = 0.6976) (Figure 4 and (*p* < 0.0001) linear regression (R^2^ = 0.7064) (Figure 5), respectively.

**Figure 6:**
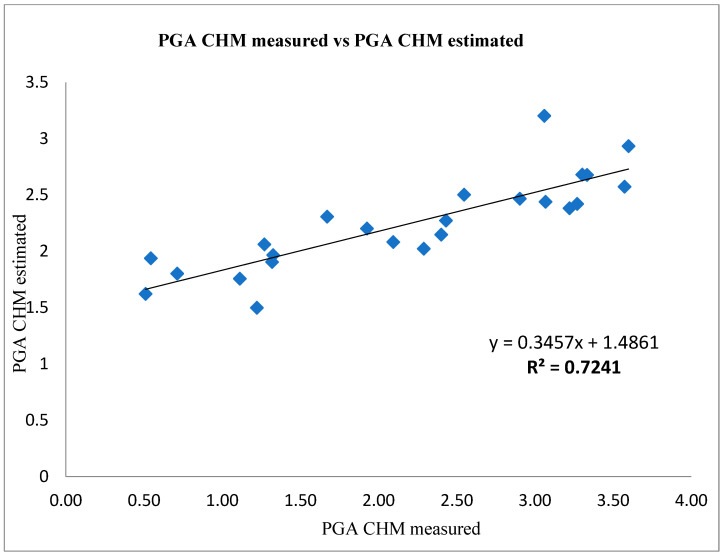
Measured vs. estimated CHM PGA with NDVI values > 0.30 of Tonda di Giffoni, using CHMe = 0.3457 × CHMm + 1.4861 equation; *p* < 0.001.

In the analysis of the predictive ability to estimate the relationship using 25 values measured vs. estimated PGA CHM by NDVI values > 0.30 of Tonda di Giffoni, using the CHMe = 0.3457 × CHMm + 1.4861 equation showed a statistically significant (*p* < 0.001) and high linear regression (R^2^ = 0.7241) (Figure 6).

**Figure 7:**
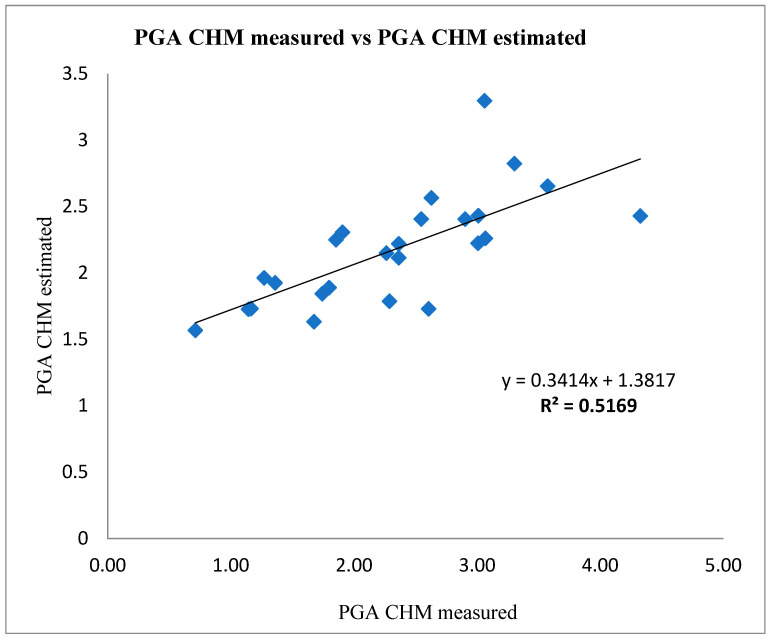
Measured vs. estimated CHM PGA with NDVI values > 0.35 of Tonda di Giffoni, using CHMe = 0.3414 × CHMm + 1.3817 equation; *p* < 0.001.

In contrast, the measured PGA vs. CHM estimated with NDVI values > 0.35 of Tonda di Giffoni using the CHMe = 0.3414 × CHMm + 1.3817 equation showed statistical significance (*p* < 0.001) and low regression was (R^2^ = 0.5169) (Figure 7).

### 3.2. LAI Measurement

**Figure 8:**
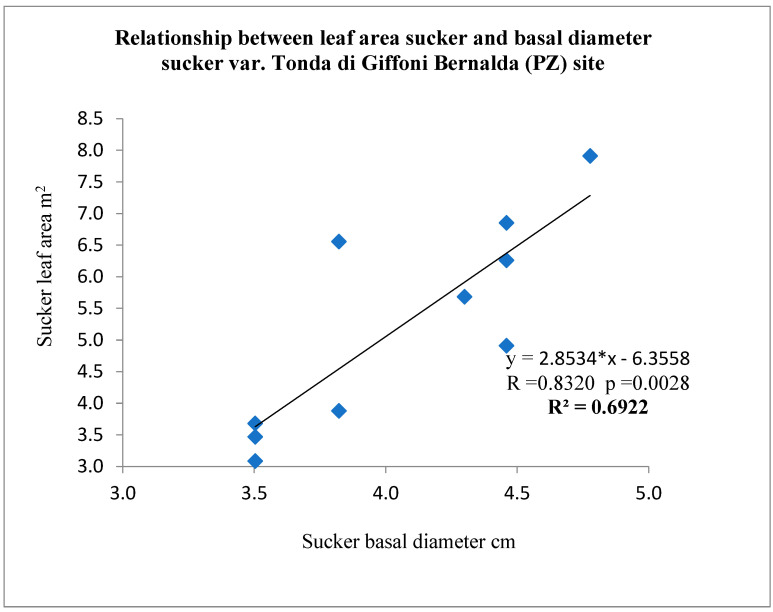
Relationship between leaf area and basal diameter of its suckers; *p* < 0.0028.

The relationship between the basal diameter of the sucker and its leaf area is shown in Figure 8. 

The linear regression obtained (R^2^ = 0.6922) was found to be statistically significant (*p* < 0.028). Thus, the relationship was used to estimate the total leaf area of the canopy. The regression between the sucker diameter and its subtended leaf area was applied to each sucker in the plant. This allowed the total area of the individual plant to be determined (Table 1).

Finally, from the ratio of the plant’s leaf area to its ground projection (PGA), the Leaf Area Index value was obtained. The results are shown in Table 1.

## 4. Discussion

The use of CHM is of paramount importance, as it allows to identify bodies above the ground level and to analyze only the vegetation of interest (in this case, the hazelnut tree canopies), and may therefore be used as a reference for the canopies obtained from NDVI.

The NDVI method allowed a satisfactory delimitation of the canopies when a threshold value > 0.30 was used (R^2^ = 0.7241) due to the high performance of the drone used, shown from the analysis of predictive ability. In contrast, the measured PGA vs. CHM estimated with NDVI values > 0.35 manifested a lower correlation (R^2^ = 0.5162) due to the exclusion of canopy pixels with NDVI values between 0.30 and 0.35.

Other authors studying different crops found similar results. Mu et al. [27], obtained time-series information using a similar method in the peach crop, based on gathered images using a digital surface model to measure the crown projection area, showing R^2^ = 0.88. In addition, in a blueberry bush [28], it showed R^2^ = 0.7. 

The ultra-high-resolution imagery collected with UAV systems helped identify and delineate each crown of trees individually from the background, according to Caruso et al. [17], even though the flight altitude was 120 m to capture the entire field. 

### LAI Measurement

The average value of LAI in our study was about nine, while the leaf area per plant was 20 m^2^. Farinelli et al. [29] Farinelli 2005 found that, in the variety Tonda di Giffoni trained with medium dense pruning and planted on a spacing of 5 m × 4.5 m, the leaf area per plant was 39.44 m^2^ for a twenty-year-old tree. However, Bignami et al. [8] showed that, in the absence of water stress, five-to-six-year-old plants of the variety Tonda Gentile Langhe spaced 4 m × 5 m and trained in a free vase, the leaf area per plant was 12.78 m^2^. The value of the leaf area index is strongly influenced by the pruning system and the water availability, as observable by different studies. Bignami and Natali [9] reported LAI values between 2.39–5.21 and leaf area per plant between 0.35–6.24 for a two-to-three-year-old tree at the end of the growing season. 

The relationship between the basal diameter of the sucker and its leaf area with R^2^ = 0.69 obtained can be compared with the results of the work conducted by Pisetta [30] on the branches of the vase bush in the variety Tonda Gentile Langhe, spaced 5 m × 5 m. The latter work showed a quadratic relationship between the diameter at the base of the branches and the subtended leaf area, with R^2^ = 0.93.

## 5. Conclusions

The irrigation of hazelnuts is a recently introduced and expanding practice and it is important to know the parameters required for sustainable irrigation scheduling. This work shows how, in complex farming systems, the use of UAVs allows to work in this direction. This aerial system, equipped with a camera with spectral sensors, allows for the calculation and monitoring of biophysical and geometric parameters of the canopy such as leaf area index (LAI) and canopy volume. 

The hazelnut orchard under study was a way to see the possibility of rapidly calculating the projected canopy in the presence of grass cover using the NDVI method. 

The limitation of this work is related to only measuring the canopy edge in the top view, which means that errors will increase with overlaps between trees. This limitation was highlighted by Mu et al. [26], in the case of characterizing the canopy of a peach tree using UAV, and by Patrick and Li [27], who selected in their study non-overlapping canopies only. 

The analysis will be extended to adult orchards to build reliable relationships between NDVI and LAI and to evaluate their spatio-temporal evolution. This extension of time makes it possible to calculate another parameter such as growth rate, which showed differences in growth over time in peach plants [26].

This study confirms that morphological traits can be extracted from hazelnut trees using high-precision UAV images. The research should be extended to the definition of UAV-assisted procedures for estimating the volume of hazelnut trees; in fact, the crown volume/PGA ratio is another frequently used character in irrigation modeling.

## Figures and Tables

**Table 1:** Estimated values of Leaf Area Index from circumference measurements and leaf area of individual suckers.

Row-No.Tree (ID Plant)	Leaves per Sucker	Average Leaf Area	Sucker Leaf Area	Mean Ø Sucker per Plant	Total Canopy Area of Single Tree	Projected Ground Area from CHM	LAI
	n.	(cm^2^)	(m^2^)	(cm)	(m^2^)	(m^2^)	
2-17	1179	53.10	6.26	4.62	27.32	3.50	7.80
11-3	1016	48.34	4.91	4.34	24.12	3.30	7.31
12-45	1375	49.84	6.85	4.86	30.06	2.00	15.03
15-14	729	47.58	3.47	2.79	12.72	1.61	7.88
17-6	1360	48.22	6.56	4.26	23.20	2.52	9.22
17-25	1307	43.49	5.68	4.58	26.86	2.85	9.43
27-34	798	38.67	3.09	3.62	15.89	1.82	8.73
34-19	753	51.52	3.88	3.86	18.63	1.16	16.01
6-6	720	51.12	3.68	2.51	9.52	2.12	4.48
17-20	1335	59.26	7.91	3.90	19.09	3.03	6.30
Mean	1057.2	49.11	5.23	3.93	20.74	2.39	9.22
±δ	284.8	5.51	1.66	0.78	6.72	0.77	3.63

## Data Availability

Not applicable.
